# Characterization of recombination features and the genetic basis in multiple cattle breeds

**DOI:** 10.1186/s12864-018-4705-y

**Published:** 2018-04-27

**Authors:** Botong Shen, Jicai Jiang, Eyal Seroussi, George E. Liu, Li Ma

**Affiliations:** 1 Department of Animal and Avian Sciences, University of Maryland, 2123 Animal Science Building, College Park, MD 20742 USA; 2 Institute of Animal Science, Agricultural Research Organization (ARO), The Volcani Center, 15159 Rishon LeTsiyon, Israel; 3 Animal Genomics and Improvement Laboratory, BARC, USDA-ARS, Beltsville, MD 20705 USA

**Keywords:** Recombination, Dairy cattle, Breed, Sex, GWAS, Pedigree

## Abstract

**Background:**

Crossover generated by meiotic recombination is a fundamental event that facilitates meiosis and sexual reproduction. Comparative studies have shown wide variation in recombination rate among species, but the characterization of recombination features between cattle breeds has not yet been performed. Cattle populations in North America count millions, and the dairy industry has genotyped millions of individuals with pedigree information that provide a unique opportunity to study breed-level variations in recombination.

**Results:**

Based on large pedigrees of Jersey, Ayrshire and Brown Swiss cattle with genotype data, we identified over 3.4 million maternal and paternal crossover events from 161,309 three-generation families. We constructed six breed- and sex-specific genome-wide recombination maps using 58,982 autosomal SNPs for two sexes in the three dairy cattle breeds. A comparative analysis of the six recombination maps revealed similar global recombination patterns between cattle breeds but with significant differences between sexes. We confirmed that male recombination map is 10% longer than the female map in all three cattle breeds, consistent with previously reported results in Holstein cattle. When comparing recombination hotspot regions between cattle breeds, we found that 30% and 10% of the hotspots were shared between breeds in males and females, respectively, with each breed exhibiting some breed-specific hotspots. Finally, our multiple-breed GWAS found that SNPs in eight loci affected recombination rate and that the *PRDM9* gene associated with hotspot usage in multiple cattle breeds, indicating a shared genetic basis for recombination across dairy cattle breeds.

**Conclusions:**

Collectively, our results generated breed- and sex-specific recombination maps for multiple cattle breeds, provided a comprehensive characterization and comparison of recombination patterns between breeds, and expanded our understanding of the breed-level variations in recombination features within an important livestock species.

**Electronic supplementary material:**

The online version of this article (10.1186/s12864-018-4705-y) contains supplementary material, which is available to authorized users.

## Background

In eukaryotes, meiotic recombination promotes genetic variation by reciprocal exchange of genetic materials between maternal and paternal homologs and introduction of new combinations of genetic variants into future generations. Aberrant meiotic recombination can cause aneuploidy and often lead to deleterious outcomes [1, 2]. As a fundamental biological process, the genetic mechanisms of meiotic recombination are conserved across all eukaryotic species [3].

Humans and chimpanzees show little conservation on the high-resolution recombination landscape, suggesting a rapid evolution of recombination among species [4, 5]. Pedigree-based studies have discovered considerable variation in recombination rate in humans and mice [6, 7]. Sex-specific recombination maps have been generated in several mammalian species with the sex difference in recombination confirmed. Females have a higher recombination rate than males in many mammals, including human [8, 9], mouse [10], dog [11], pig [12], and red deer [13]. However, males have more recombination events per meiosis in sheep [14] and cattle [15, 16]. Despite the extensive variation in recombination between species and sexes, only a few studies have examined the effect of within-species variation on recombination landscape, mostly in humans [17, 18].

Genome-wide association studies (GWAS) have identified genetic variants associated with recombination features in human [19, 20], mouse [21], cattle [15, 22], and sheep [23] studies. Several genes, including *RNF212*, *CPLX1* and *PRDM9*, were associated with individual-level recombination rate across multiple species. Recombination events are more likely to occur in short genomic regions known as recombination hotspots [21]. Many studies have shown that localization of recombination hotspots is associated with the *PRDM9* gene in mammals, with the exception of canids that carry a non-functional copy of *PRDM9* [24]. Moreover, the fast-evolving *PRDM9* gene is known as a speciation gene that causes hybrid sterility in multiple mouse subspecies [25]. Taken together, these studies suggest the existence of genetic basis of recombination that may facilitate a quick response to selection in a short period of time. However, recent simulation-based studies by Battagin et al. highlighted the difficulty of manipulating recombination rate on selective breeding in livestock populations, especially for polygenic traits [26, 27].

The U.S. dairy population consists of many cattle breeds, with the most popular ones being Holstein, Jersey, Brown Swiss, and Ayrshire [28]. These four dairy breeds were brought to the U.S. from Europe in the seventeenth century. The cattle domestication is estimated to have begun approximately 10,000 to 11,000 years ago [29], but the formation of diverse cattle breeds is far more recent. Given the fast evolution of recombination and close relationship between cattle breeds, it is questionable whether these cattle breeds exhibit different recombination landscapes. Current breeding strategies in the cattle industry heavily relied on a small number of superior bulls, which will increase inbreeding level, decrease effective population size, and reduce genetic variation in the cattle population. Recombination may be used to address these growing issues of inbreeding in the cattle industry [26, 27].

The Council on Dairy Cattle Breeding (CDCB) and USDA Animal Genomics and Improvement Laboratory (AGIL) maintain a large database for millions of cattle of different breeds with both pedigree and genotype information. This existing database provides a unique opportunity to study recombination features across multiple breeds but within the bovine species. Using this large database, we generated six breed- and sex-specific recombination maps for Jersey, Brown Swiss, and Ayrshire cattle in two sexes, respectively. Comparing to the previously reported Holstein recombination maps, we examined the similarities and differences between eight recombination maps and documented significant breed- and sex-specific recombination hotspot regions, revealing both broad- and fine-scale recombination features that differed between cattle breeds. Finally, we performed GWAS analyses of recombination features to understand the genetic basis of recombination in multiple cattle breeds and in two sexes.

## Results

### Identification of crossover events using genotyped cattle pedigree

Using a similar approach to our previous studies in Holstein cattle [15, 30], we constructed three-generation families that included an offspring, parents, and grandparents from large pedigrees of three dairy cattle breeds. Within a three-generation family, we phased the SNP genotypes of the offspring and parents based on parental genotype data. By comparing phased genotypes between a sire-offspring or dam-offspring pair, we inferred paternal or maternal crossover events. We used a total of 144,079 genotyped individuals across three dairy cattle breeds, with Jersey accounting for 83.4%, Brown Swiss 13.4%, and Ayrshire 3.2% of the data, respectively (Table 1). In total, we identified over 3.4 million crossover events for Jersey, 0.41 million for Brown Swiss, and 51,982 for Ayrshire (Table 2). Holstein data were published previously and included for comparison purposes [15]. To ensure data quality, we excluded the X chromosome and used the USDA-AGIL SNP coordinates that removed likely errors in the UMD 3.1 Bovine genome assembly [31, 32]. To calculate recombination rate between SNPs, we assigned a crossover event evenly to all consecutive SNP intervals between two informative SNPs and generated breed- and sex-specific recombination maps for Jersey, Brown Swiss, and Ayrshire in the two sexes using 58,982 autosomal SNPs (Additional file 1). These breed- and sex-specific recombination maps will be useful in various genetic studies of cattle of different breeds, including imputation, selection signature detection, genomic selection, and genetic simulation studies.

**Table 1:** Number of genotyped three-generation families by breed, sex, and SNP density across three dairy cattle breeds

Breed	Male		Female		Total
	< 50 K SNP	≥50 K SNP	< 50 K SNP	≥50 K SNP	
Jersey	8409	9409	99,487	2787	120,092 (83.4%)
Brown Swiss	1441	14,191	3241	382	19,255 (13.4%)
Ayrshire	160	1355	2049	1168	4732 (3.2%)
Total	34,965(24.3%)		109,114(75.7%)		144,079

**Table 2:** Number of meiosis and crossover, and genome-wide recombination rate by breed and sex

Breed	Crossover		Meiosis		Recombination Rate	
	Male	Female	Male	Female	Male	Female
Jersey	> 2.3 M	> 0.7 M	108,163	37,008	23.7	22.2
Brown Swiss	328,653	9804	13,556	436	24.2	22.5
Ayrshire	40,161	11,821	1620	526	24.8	22.5

### Global recombination patterns in multiple cattle breeds and two sexes

**Fig. 1:**
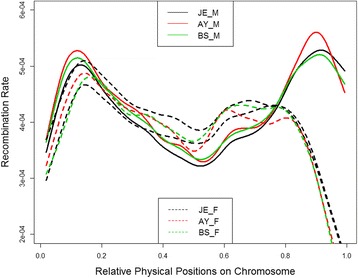
Distribution of male and female recombination rates along a chromosome in Jersey, Ayrshire, and Brown Swiss cattle. The relative physical position for each SNP interval on a chromosome was calculated by standardizing the original physical position by the chromosome length: a value of zero corresponds to the beginning of a chromosome and a value of one corresponds to the end. Solid lines: males, dotted lines: females. The smooth-spline model was fitted across all 29 autosomes

To account for different SNP densities, we only included those crossovers identified from 50 K SNP data in the comparisons. In Jersey cattle, the average number of crossovers per meiosis was 23.7 and 22.2 respectively for males and females. This is consistent with the previously reported higher recombination rate in bulls than in cows in Holstein cattle [15, 22, 30]. This male-biased recombination rate was also confirmed in the other two breeds: an average of 24.4 male and 22.5 female crossovers per meiosis in Brown Swiss, and 24.8 and 22.5 in Ayrshire (Table 2). Compared between breeds, Jersey cattle had slightly less crossovers than other breeds in both sexes (3% ~ 5% less in males and 1.3% ~ 2.3% in females). To visualize the breed-specific recombination patterns along the genome, we generated smooth-spline plots of recombination rate versus chromosomal location in three breeds and two sexes, respectively (Fig. 1). Overall, cattle recombination rate along the genome exhibited larger variations between sexes than between cattle breeds. All the cattle autosomes are acrocentric, with centromeres at the beginning and telomeres to the end of chromosomes [33]. Males and females showed similar recombination patterns across the chromosomes with large sex differences near the end of chromosomes (telomeres in cattle). All three breeds showed a similar trend across the chromosomes: males had a considerably higher recombination rate near the end of chromosomes (telomeres in cattle), a lower recombination rate in the middle of chromosomes, and a slightly higher rate at the beginning of chromosomes (centromeres and centromeres in cattle). In fact, the cattle centromeres are located almost at the extremity of the chromosomes, so we probably observed a mixed effect from both centromere and telomere at the beginning of chromosomes. In both sexes, Jerseys showed the lowest recombination rates along the chromosomes, except for the telomeric regions. We also calculated correlations in recombination maps between four cattle breeds in two sexes (Table 3). Holstein and Jersey had the highest correlations in both males and females, while Brown Swiss and Ayrshire showed the lowest correlations in the two sexes. However, when we used the same number of individuals to construct recombination maps in these cattle breeds, we observed similar correlations among breeds (Additional file 2), suggesting a conserved global recombination map between breeds and that the observed different correlations between breeds are likely due to different sample sizes.

**Table 3:**
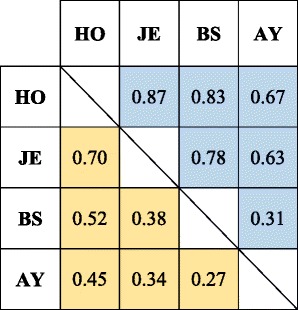
Correlation coefficient between recombination maps of four cattle breeds in two sexes. Correlations in males were presented in the top-right triangle and female correlations in the bottom-left. The Holstein data have been published previously [15] and are included for comparison purposes

### Regional recombination patterns in multiple cattle breeds and two sexes

**Fig. 2:**
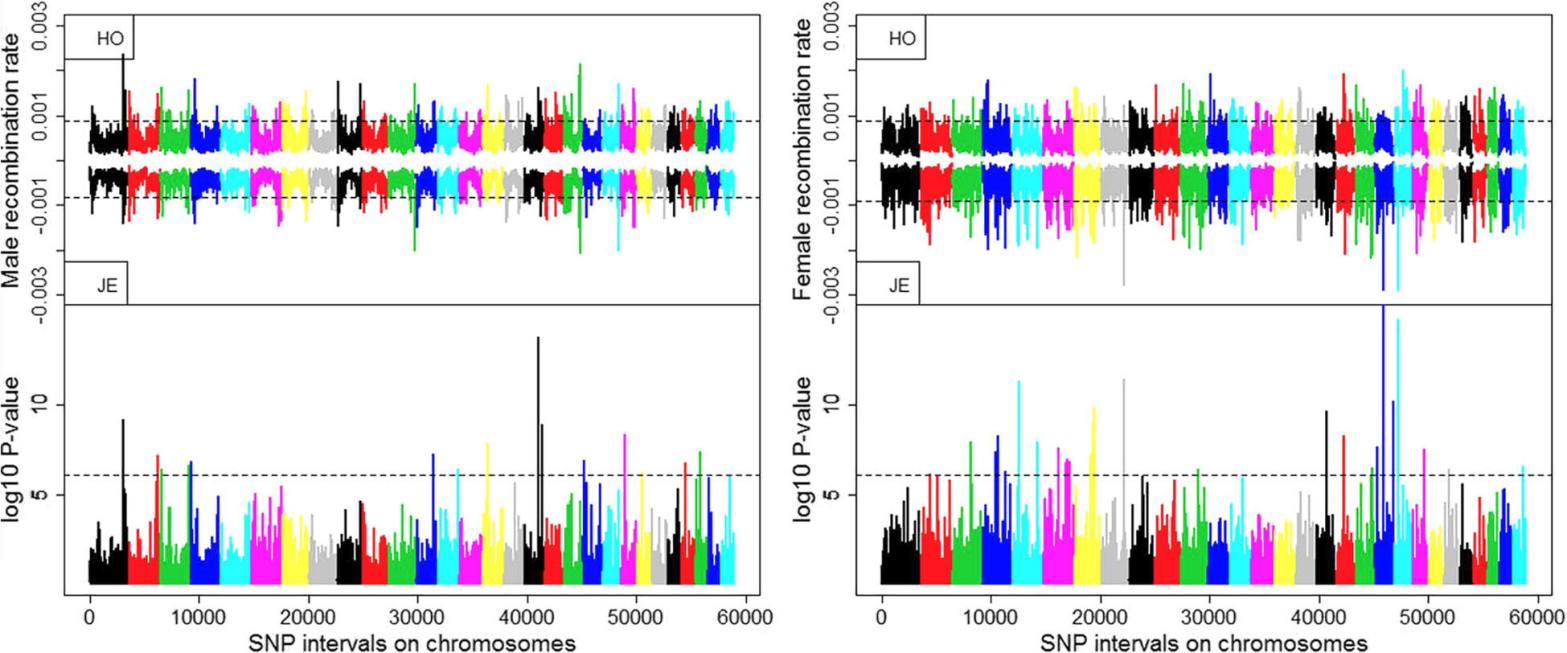
Breed-specific recombination locations between Holstein and Jersey in males (left) and females (right). For each panel, recombination rates in each SNP intervals of two groups were shown in the top half and corresponding *P*-values were shown in bottom. Different colors were used to distinguish the 29 chromosomes. The dash line shows the significance level of *P*-value < 8.3 × 10^− 7^ after Bonferroni correction. The Holstein data have been published previously [15] and are included for comparison purposes

To identify breed-specific locations of recombination, we applied a Chi-square test to find SNP intervals with significantly different recombination rate between four dairy breeds in two sexes. Using a genome-wide significance level of *P*-value < 8.3 × 10^− 7^ after Bonferroni correction, we identified 21 SNP intervals with different recombination rate between Holstein and Jersey in males and 43 such SNP intervals in females (Fig. 2). The most Holstein favored recombination interval (most different between Holstein and other breeds but more frequent in Holstein) was located on chromosome 22, where Holstein, Jersey, Ayrshire and Brown Swiss respectively has a recombination rate of 0.0008, 0.0003, 0.0002, and 0.0004, showing a 3.9-fold increase between Holstein and Jersey, 3.2-fold increase between Holstein and Ayrshire, and 2-fold increase between Holstein and Brown Swiss. However, we didn’t find any intervals with different recombination rate between other pairs of cattle breeds, mainly due to the small sample sizes of Brown Swiss and Ayrshire data. More detailed differences in recombination pattern between cattle breeds were revealed as we zoomed into the regional recombination maps of the four cattle breeds (Additional file 3).

### Sharing of hotspot regions between cattle breeds in two sexes

To further characterize local recombination patterns, we tentatively defined hotspot regions as SNP intervals with recombination rate > 2.5 standard deviations above the mean. We herein used the term “hotspot region” instead of “hotspot” because our SNP intervals were much larger (average 44 kb) than typical human or mouse recombination hotspots (a few kb or smaller). In males, we identified 1378, 1295, and 1317 hotspot regions for Jersey, Brown Swiss, and Ayrshire, respectively. Similar numbers of hotspot regions were found in females: 1289, 1421, and 1327 for the three breeds, respectively. A total of 256 (20%) hotspot regions were shared between sexes in the Jersey cattle, but this number dropped to 128 (9.4%) and 115 (8.8%) for Ayrshire and Brown Swiss, respectively. This relatively low sharing of hotspot regions between sexes was consistent with the sex differences we observed in global recombination patterns.

To evaluate breed-specific distributions of recombination hotspots, we compared hotspot regions across three cattle breeds in two sexes. In males, Jersey, Ayrshire, and Brown Swiss each had 394, 480, and 708 unique hotspot regions, with 233 hotspots shared by all breeds. In addition to the 233 common hotspots, Jersey and Ayrshire shared 40 hotspot regions, Jersey and Brown Swiss shared 102 hotspot regions, and Ayrshire and Brown Swiss shared 65 hotspot regions. In females, Jersey, Ayrshire, and Brown Swiss each had 714, 914, and 1092 unique hotspot regions, with 44 hotspot regions shared by all breeds. We observed the same trend of hotspot sharing in cows as in bulls: excluding the common hotspots, Jersey and Ayrshire shared 65 hotspot regions, Jersey and Brown Swiss shared 46 hotspot regions, and Ayrshire and Brown Swiss shared 47 hotspot regions. These hotspot sharing results were consistent with the phylogenetic relationships that were reported in diverse cattle populations based on 50 K SNP chips [29].

### GWAS of genome-wide recombination rate in multiple breeds and two sexes

To reduce biases caused by SNP density differences, recombination rate was adjusted by the number of informative SNP markers in the three-generation families. Using the adjusted genome-wide recombination rate as phenotype, our GWAS analysis included 2237, 1217, and 340 bulls and 18,029, 817, and 791 cows for Jersey, Brown Swiss, and Ayrshire, respectively. Compared to the previous GWAS in Holsteins [15], this study had smaller sample sizes but more cattle breeds. We used a genome-wide significance level of *P*-value < 7.3 × 10^− 7^ after Bonferroni correction.

**Fig. 3:**
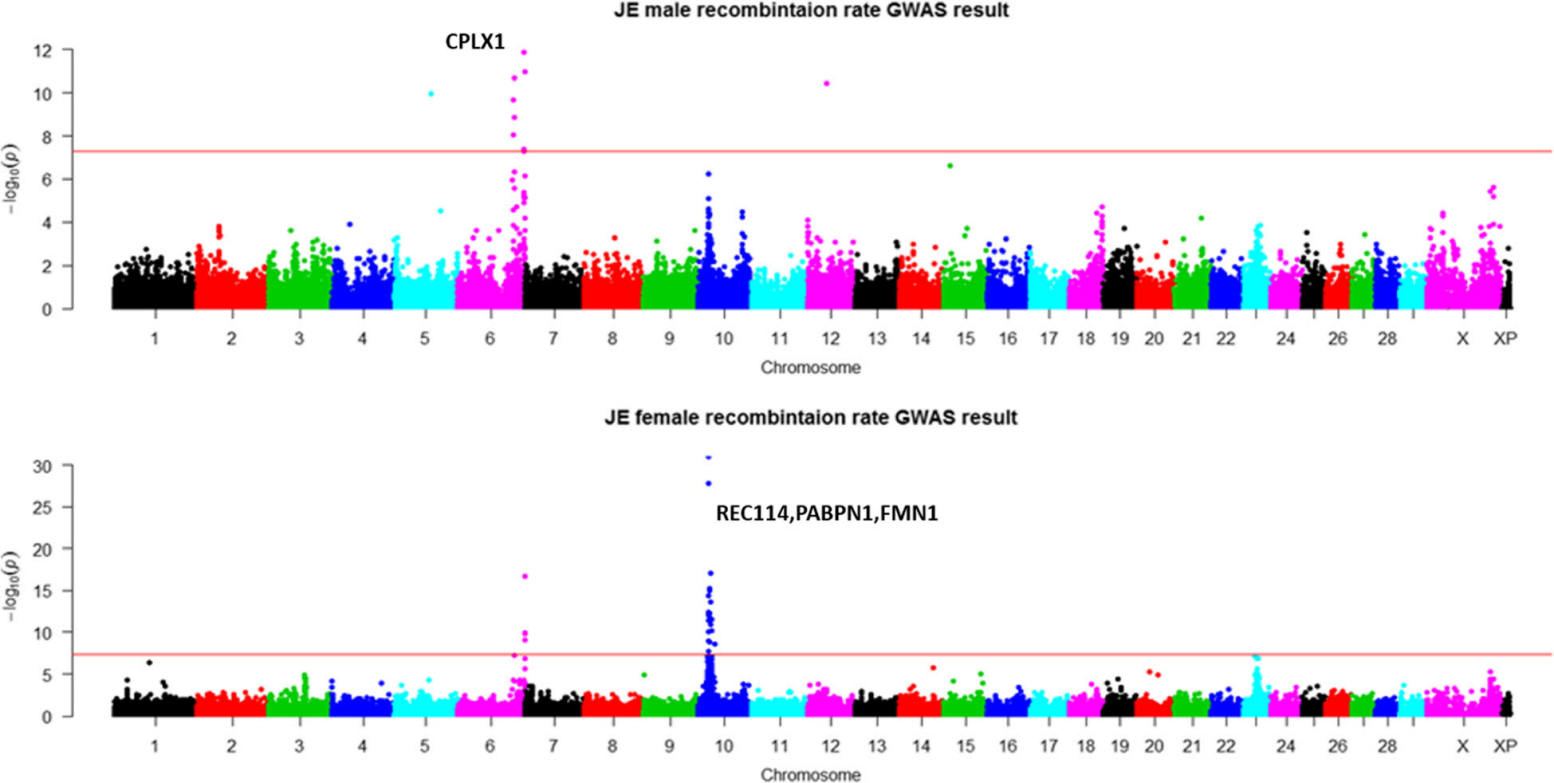
Manhattan plot of the GWAS of genome-wide recombination rates for Jerseys in two sexes. Top: Males; Bottom: Females. Different colors were used to distinguish the 29 chromosomes. The genome-wide significance level of 7.3 × 10^− 7^ was shown by the horizontal dotted line

We successfully validated our previous GWAS results reported in Holstein cattle, including four and seven loci that were significantly associated with male and female recombination rates, respectively (Table 4). While the previous study identified three associated loci shared between sexes, this study found two additional shared loci between sexes, one on chromosome 1 near the *PRDM9* gene and the other on chromosome 3 near *MSH4*. In the previous study, these two loci were associated only with female recombination rate but not in males. In total, five of the seven associated loci were shared between sexes with the same effect direction in the Holstein cattle (Table 4). In Jerseys, although the genome-wide recombination rate was lower compared to Holsteins, we found two loci significantly associated with recombination rate on chromosome 6 and chromosome 10 (Fig. 3). The top candidate genes involved in the two loci were *CPLX1* and *REC114*. The *CPLX1* gene has been reported to be associated with recombination rate in human and cattle studies [6, 15]. *REC114* is involved in DNA double-strand break formation during meiosis [34]. GWAS for Ayrshire and Brown Swiss found no associations passing the genome-wide significance threshold, likely due to the small sample sizes and low statistical power. However, most of the associated loci in Holstein and Jersey showed the same effect direction in Ayrshire and Brown Swiss, and many of them reached nominal significance levels (Table 4). Considering the different power and close relatedness between these dairy cattle breeds, these shared associations likely indicate common genetic variations underlying recombination between the four cattle breeds.

**Table 4:** Top SNPs associated with genome-wide recombination rate in two sexes and four cattle breeds. The Holstein data have been published previously [15] and are included for comparison purposes

SNP	Chr	Pos	Holstein				Jersey				Brown Swiss				Ayrshire			
			Male		Female		Male		Female		Male		Female		Male		Female	
			Beta	*P*	Beta	*P*	Beta	*P*	Beta	*P*	Beta	*P*	Beta	*P*	Beta	*P*	Beta	*P*
ARS-BFGL-NGS-83544	1	158,140,250	0.57	1.4 × 10^−12^	0.51	5.0 × 10^−31^	0.53	0.30	0.24	0.46	0.80	6.5 × 10^−4^	0.58	5.0 × 10^− 3^	0.75	0.05	0.37	2.0 × 10^−3^
Hapmap58808-rs29017431	3	52,612,595	0.52	1.5 × 10^−7^	0.36	1.8 × 10^−11^	0.14	4.9 × 10^−2^	0.32	1.3 × 10^−5^	0.57	4.6 × 10^−5^	0.15	4.8 × 10^−1^	0.12	0.07	0.27	0.74
BovineHD0600030770	6	109,176,815	−0.78	2.8 × 10^−39^	−0.38	4.4 × 10^− 35^	− 0.64	2.1 × 10^−11^	− 0.32	6.2 × 10^− 8^	− 0.73	4.3 × 10^−5^	− 0.15	1.8 × 10^− 1^	− 0.67	5.0 × 10^− 5^	− 0.26	2.5 × 10^− 1^
ARS-BFGL-NGS-117763	6	129,108,015	0.92	1.3 × 10^− 47^	0.57	3.0 × 10^− 63^	0.61	1.1 × 10^− 11^	0.47	2.1 × 10^− 17^	0.64	2.7 × 10^−4^	0.65	2.8 × 10^− 1^	0.58	5.4 × 10^− 3^	0.49	0.85
ARS-BFGL-BAC-10975	10	21,225,382	− 0.74	1.4 × 10^− 32^	− 0.42	3.8 × 10^− 38^	− 0.35	1.3 × 10^− 3^	− 0.46	8.1 × 10^− 13^	− 0.30	7.6 × 10^−2^	− 0.32	5.2 × 10^− 2^	−0.31	0.08	−0.39	0.66
BovineHD1000009771	10	29,619,086	−0.03	0.6	0.34	4.5 × 10^− 11^	−0.17	1.3 × 10^−2^	0.31	5.1 × 10^−6^	0.03	0.85	0.83	1.4 × 10^−2^	−0.09	0.87	0.39	0.06
BTA-78285-no-rs	10	86,717,378	−0.57	1.9 × 10^−20^	− 0.41	8.0 × 10^−37^	−0.23	1.9 × 10^− 2^	− 0.14	2.0 × 10^− 2^	− 0.48	5.8 × 10^− 3^	− 0.33	9.4 × 10^− 2^	− 0.26	4.8 × 10^− 3^	− 0.37	0.56
ARS-BFGL-NGS-23945	26	31,439,013	−0.15	1.6 × 10^− 2^	− 0.18	2.7 × 10^−8^	0.007	0.94	− 0.18	4.2 × 10^− 3^	− 0.13	0.57	−0.80	0.78	0.08	0.18	−0.11	0.46

### GWAS of recombination hotspot usage in two sexes

**Fig. 4:**
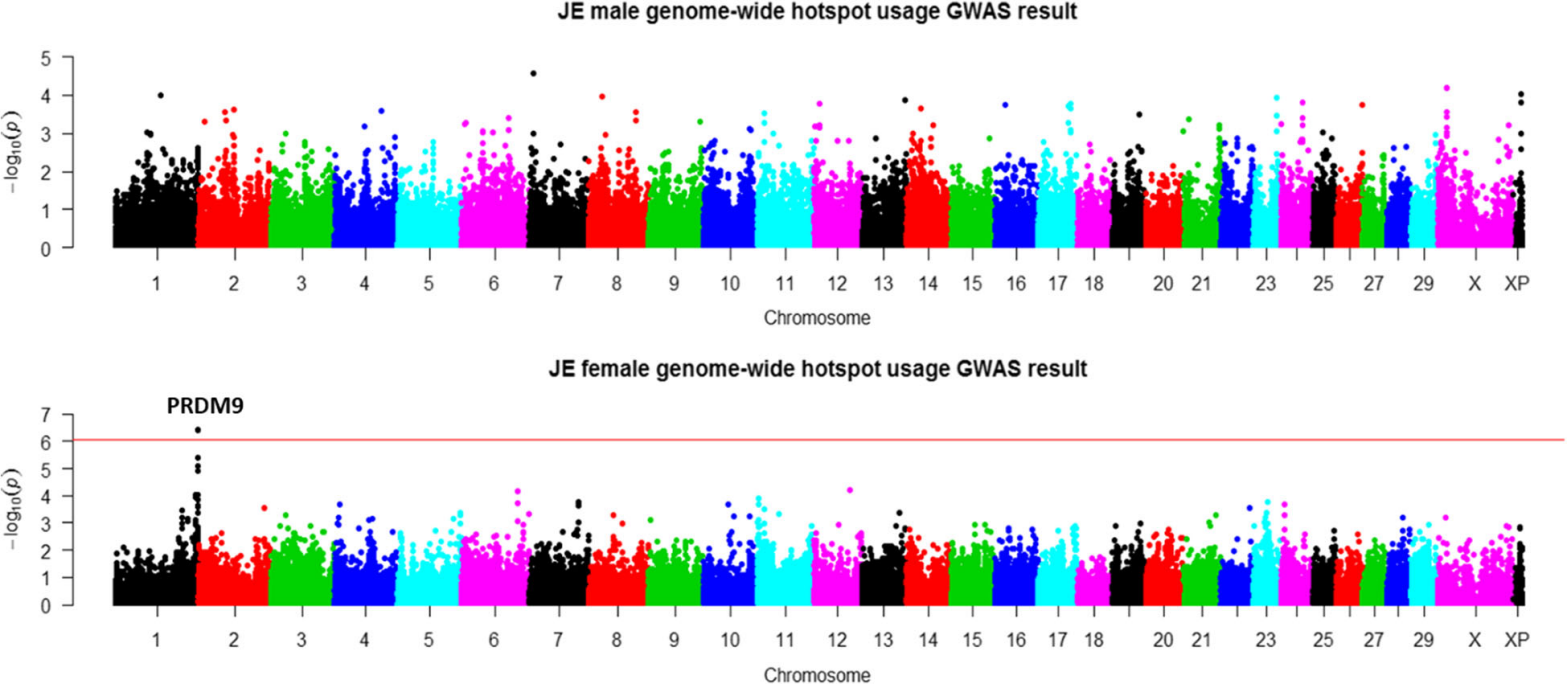
Manhattan plot of GWAS of hotspot usage for Jersey cattle in two sexes. Top: Males; Bottom: Females. Different colors were used to distinguish the 29 chromosomes. The genome-wide significance level of 7.3 × 10^− 7^ was shown by the horizontal dotted line. USDA-AGIL SNP coordinates were used for plotting, which placed *PRDM9*-linked SNPs to the end of Chromosome 1

Using the hotspot regions identified in each of the four cattle breeds, we measured hotspot usage as the proportion of recombination occurred in hotspot regions for individual animals. To increase accuracy of this measurement, we used only those three-generation families that were genotyped by 50 K or higher density SNP chips. In males, the GWAS sample sizes were 923, 728 and 994 for Jersey, Ayrshire, and Brown Swiss, respectively. The female sample sizes were 986, 343, and 165 for the three breeds, respectively. Consistent with previous GWAS studies in Holstein, we identified a single locus near *PRDM9* to be associated with hotspot usage in Jersey cattle, indicating hotspot usage to be a less polygenic trait compared to recombination rate (Fig. 4). In Holsteins, the top associated SNP ARS-BFGL-NGS-83544 was located downstream of *PRDM9* (Table 5). In Jersey cattle, we found the same top SNP associated with hotspot usage in females but not in males (Fig. 4). Although this association was not confirmed in Ayrshire or Brown Swiss, the effect direction was consistent across all four cattle breeds in both sexes (Table 5).

**Table 5:** Top SNP associated with hotspot usage in two sexes and four cattle breeds. The Holstein data have been published previously [15] and are included for comparison purposes

SNP	Chr	Pos	Holstein				Jersey				Brown Swiss				Ayrshire			
			Male		Female		Male		Female		Male		Female		Male		Female	
			Beta	*P*	Beta	*P*	Beta	*P*	Beta	*P*	Beta	*P*	Beta	*P*	Beta	*P*	Beta	*P*
ARS-BFGL-NGS-83544	1	158,140,250	−0.01	2.4 × 10^−22^	−0.01	2.8 × 10^−57^	− 0.005	3.1 × 10^−3^	−0.005	3.7 × 10^−7^	− 0.003	0.4	− 0.005	0.5	− 0.002	0.74	− 0.002	0.6

## Discussion

In this study, we took advantage of the large-scale pedigree data maintained by the Council of Dairy Cattle Breeding (CDCB) to characterize recombination landscapes of three dairy cattle breeds and to provide comprehensive comparisons between breed-specific recombination maps in two sexes. Our study confirmed the sex difference in recombination rate in three cattle breeds with male recombination map being > 10% longer than that of the females. The main sex differences in cattle recombination were found near the telomeres, which is consistent with other mammalian species. Both males and females had a decreased recombination rate around the center of chromosomes in cattle, possibly due to crossover interference [30].

While each dairy cattle breed had specific features in the distribution of recombination rate and hotspot regions, the four breeds showed similar global recombination patterns across the genome, Since the Holstein breed had the largest sample size and most accurate recombination maps, the Holstein recombination map can be used as an alternative when studying other dairy cattle breeds.

Consistent with the results from other dairy traits, we observed shared GWAS signals across multiple dairy cattle breeds. The GWAS of recombination rate in Jersey cattle found two associated loci on chromosome 6 and chromosome 10, which are also the top associations identified in the Holstein cattle. The GWAS of hotspot usage in Jersey identified a significant association near the *PRDM9* gene, although the top SNP is different from the top associated SNP in Holstein cattle. Collectively, the multiple-breed GWAS confirmed previously reported loci in Holsteins, indicating a shared genetic basis of recombination between dairy cattle breeds.

## Conclusions

In sum, we characterized cattle recombination landscape in three dairy cattle breeds and generated six breed- and sex-specific recombination maps that will be useful in genetic studies of different cattle breeds. While these breed-specific recombination maps were similar in the genome-wide scale, we discovered breed specific recombination hotspot regions and identified genetic variants associated with recombination features across multiple cattle breeds. These results provided useful insights into the genetic mechanism and evolution of recombination between cattle breeds and within an important livestock species.

## Methods

### Genotype and pedigree data from multiple cattle breeds

The genotype and pedigree data were extracted from the large U.S dairy genomics database maintained at CDCB (https://www.uscdcb.com/) and USDA AGIL (https://www.ars.usda.gov/northeast-area/beltsville-md/beltsville-agricultural-research-center/agil/). These data were mostly collected from the dairy industry. We included a total of 120,092 Jersey, 19,255 Brown Swiss, and 4732 Ayrshire cattle that have both genotype and pedigree data (Table 1). The Holstein data (*n* = 929,835) has been published previously but included for comparison purposes [15].

### Estimation of recombination rate in cattle pedigree

We used a similar approach that was described in more details previously in Holstein cattle [15]. First, we extracted three-generation families from the pedigree of Holstein, Jersey, Ayrshire, and Brown Swiss cattle. We required each three-generation family to have an offspring (first generation), at least one parent (second generation), and at least one grandparent (third generation) to be genotyped. We then phased the two haplotypes of an animal (second or third generation) based on the parental genotypes, and crossover locations were identified by comparing either a paternal or maternal haplotype of an offspring (third generation) to its corresponding parent’s two haplotypes (second generation). Based on the location of a crossover, a recombination event was assigned to an interval flanked by two informative SNPs (phased heterozygote SNPs in the second generation). To construct recombination maps, we estimated recombination rate between consecutive SNPs as the average number of crossovers per meiosis by evenly assigning a crossover event to all SNP intervals between two informative SNPs. To ensure high-quality recombination maps, we only used those three-generation families genotyped by at least 50 K SNP chips. For quality control purposes, we removed animals that had more than 45 crossover events genome-wide, based on the distribution of crossover events across all animals. The X chromosome was excluded from all analyses due to the poor quality of the assembly of chromosome X in the current bovine genome assembly.

### Global and local comparisons of recombination maps between breeds

To show the global distribution of recombination rates along the chromosomes, we adopted a smooth spline model of recombination rates against relative physical locations of chromosomes using the smooth.spline function implemented in R 3.2.4 [35]. We divided the recombination data into subgroups based on breed and sex to evaluate the patterns of recombination map in each subgroup. To identify breed-specific recombination hotspot regions, we locally compared recombination rate in a SNP interval between breed pairs across four cattle breeds. We applied a Chi-square test to determine if the proportion of crossover events in a SNP interval per meiosis is different between two cattle breeds. There were unequal numbers of animals for the four breeds due to different popularity in the dairy industry, which may reduce the power of the Chi-square test in the non-popular breeds.

### GWAS of genome-wide recombination rate and hotspot usage using a linear mixed model

From each three-generation families, we estimated the total number of crossover events per meiosis of the sire or dam (second generation). We adjusted the number of crossover events by SNP density and the number of informative markers (phased heterozygote SNPs) of each animal, and used the adjusted numbers of crossovers for further analyses. A linear model was fitted using the crossover number as the response variable and SNP density, number of informative markers in the parent, and number of informative markers in the offspring as explanatory variables. We then calculated the adjusted number of crossovers as the residual from the linear model. Each sire or dam may have multiple crossover measurements if they had multiple offspring, in which case we calculated the average adjusted crossovers as the phenotype of recombination rate. Hotspot regions were tentatively defined as SNP intervals with recombination rate > 2.5 standard deviations above the mean. Hotspot usage was calculated as the proportion of crossover events that occurred in the hotspot regions per meiosis. The average of multiple hotspot usages was used when an individual has more than one meioses typed and therefore multiple measurements of hotspot usage available. To ensure data quality for GWAS, we only included three-generation families where all animals were genotyped by at least 50 K SNP chips. Using genome-wide recombination rate and hotspot usage as phenotypes, we tested for the association between a phenotype and each SNP by a linear mixed model. The model equation was fitted as following,

$$ \mathrm{y}=\mathrm{Xg}+\mathrm{Za}+\mathrm{e} $$

where **y** refers to the phenotypic value of individuals, **X** is the design matrix of the fixed effects **g**, which include a population mean and the additive effect of the candidate SNP. **Z** is a design matrix for the random animal effect **a**, and **e** represents residuals. The MMAP software was used for all GWAS analyses [36].

## Additional files

Additional file 1: Eight breed- and sex-specific recombination maps for Holstein, Jersey, Brown Swiss, and Ayrshire in the two sexes. The columns are SNP name, chromosome number, base pair position, recombination rates between current and preceding SNPs in Holstein females, Holstein males, Jersey females, Jersey males, Brown Swiss females, Brown Swiss males, Ayrshire females, and Ayrshire males, respectively. The Holstein data have been published previously [15] and are included for comparison purposes. (RMAP 7191 kb)

Additional file 2: Correlation between recombination maps of four cattle breeds using the same sample size. The average correlations were calculated from 1000 repeatedly random samples. Each random sample has the same number of meioses across four cattle breeds. Correlations in males were presented in the top-right triangle and female correlations in the bottom-left. The Holstein data have been published previously [15] and are included for comparison purposes. (DOCX 14 kb)

Additional file 3: Example regions showing different recombination patterns between four cattle breeds. Top 2: males; Bottom 2: females. The Holstein data have been published previously [15] and are included for comparison purposes. (DOCX 183 kb)

## Abbreviations

AY: Ayrshire
BS: Brown Swiss
Chr: Chromosome
GWAS: Genome-wide association study
HO: Holstein
JE: Jersey
QTL: Quantitative trait locus
SNP: Single nucleotide polymorphism
