# Supplementary material for: Characterization of recombination features and the genetic basis in multiple cattle breeds
Source: BMC Genomics. 2018 Apr 27;19:304. doi: 10.1186/s12864-018-4705-y (PMC5923192; doi:10.1186/s12864-018-4705-y)
Supplement: Supplementary file 2 — Correlation between recombination maps of four cattle breeds using the same sample size. The average correlations were calculated from 1000 repeatedly random samples. Each random sample has the same number of meioses across four cattle breeds. Correlations in males were presented in the top-right triangle and female correlations in the bottom-left. The Holstein data have been published previously [15] and are included for comparison purposes. (DOCX 14 kb) [file 12864_2018_4705_MOESM2_ESM.docx]

**Additional File 2. Correlation between recombination maps of four cattle breeds using the same sample size.** The average correlations were calculated from 1000 repeatedly random samples. Each random sample has the same number of meioses across four cattle breeds. Correlations in males were presented in the top-right triangle and female correlations in the bottom-left. The Holstein data have been published previously [15] and are included for comparison purposes.

|  | **HO** | **JE** | **BS** | **AY** |
| --- | --- | --- | --- | --- |
| **HO** |  | 0.51 | 0.52 | 0.51 |
| **JE** | 0.22 |  | 0.52 | 0.49 |
| **BS** | 0.23 | 0.24 |  | 0.50 |
| **AY** | 0.25 | 0.23 | 0.25 |  |
