# Supplementary material for: Characterization of recombination features and the genetic basis in multiple cattle breeds
Source: BMC Genomics. 2018 Apr 27;19:304. doi: 10.1186/s12864-018-4705-y (PMC5923192; doi:10.1186/s12864-018-4705-y)

**Additional File 3: Example regions showing different recombination patterns between four cattle breeds.** Top 2: males; Bottom 2: females. The Holstein data have been published previously [15] and are included for comparison purposes.


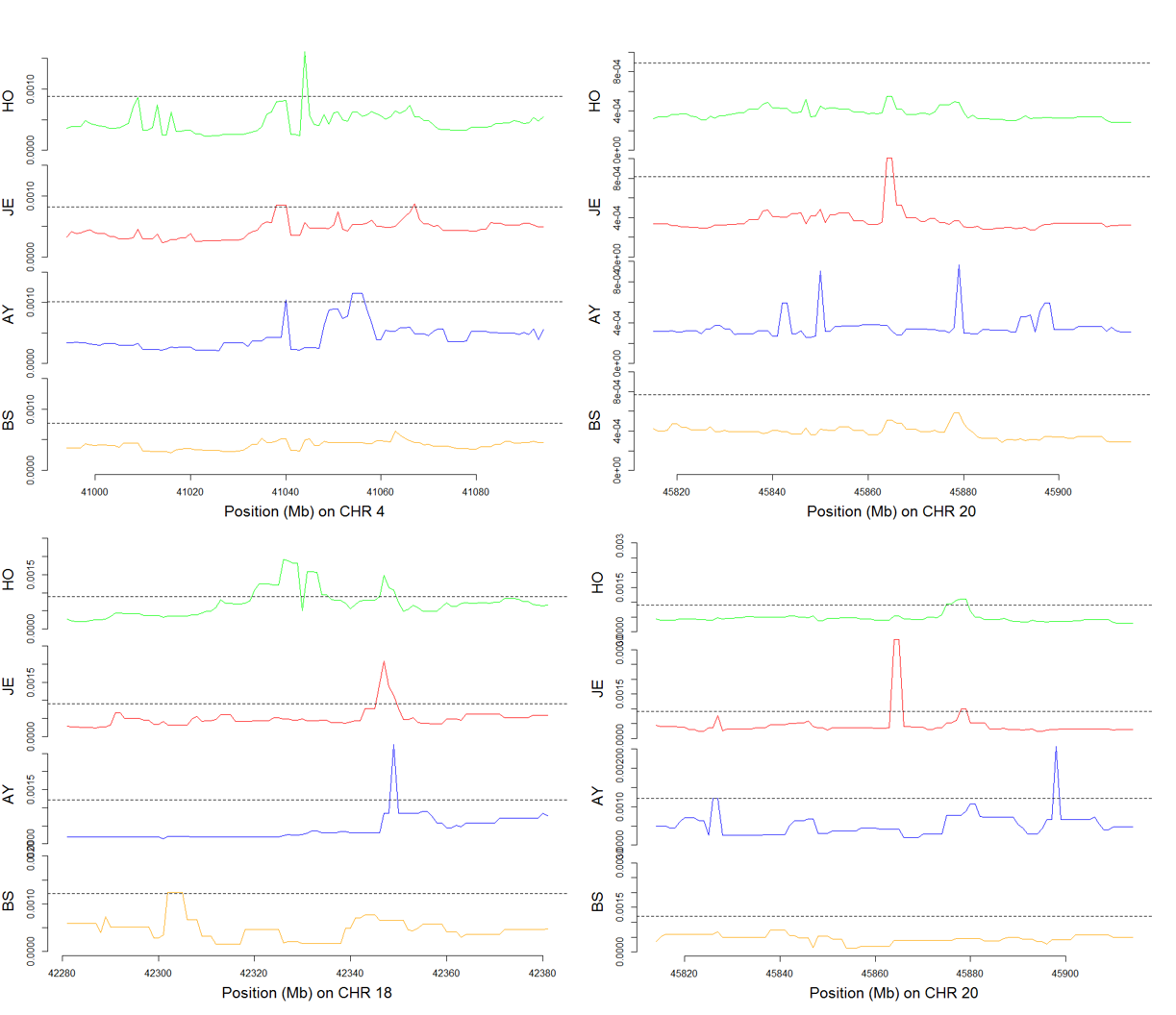

Supplement: Supplementary file 3 — Example regions showing different recombination patterns between four cattle breeds. Top 2: males; Bottom 2: females. The Holstein data have been published previously [15] and are included for comparison purposes. (DOCX 183 kb) [file 12864_2018_4705_MOESM3_ESM.docx]
